# A networked station system for high-resolution wind nowcasting in air traffic operations: A data-augmented deep learning approach

**DOI:** 10.1371/journal.pone.0316548

**Published:** 2025-01-14

**Authors:** Décio Alves, Fábio Mendonça, Sheikh Shanawaz Mostafa, Diogo Freitas, João Pestana, Dinarte Vieira, Marko Radeta, Fernando Morgado-Dias

**Affiliations:** 1 University of Madeira, Funchal, Portugal; 2 Interactive Technologies Institute (ITI/LARSyS), Funchal, Portugal; 3 NOVA LINCS NOVA Laboratory for Computer Science and Informatics, Lisbon, Portugal; 4 Wave Labs, Faculty of Exact Sciences and Engineering, University of Madeira, Portugal; 5 MARE—Marine and Environmental Sciences Centre, ARNET—Aquatic Research Network, Portugal; 6 Agência Regional para o Desenvolvimento da Investigação Tecnologia e Inovação (ARDITI), Funchal, Portugal; 7 Department of Astronomy, Faculty of Mathematics, University of Belgrade, Serbia; Khalifa University, UNITED ARAB EMIRATES

## Abstract

This study introduces a high-resolution wind nowcasting model designed for aviation applications at Madeira International Airport, a location known for its complex wind patterns. By using data from a network of six meteorological stations and deep learning techniques, the produced model is capable of predicting wind speed and direction up to 30-minute ahead with 1-minute temporal resolution. The optimized architecture demonstrated robust predictive performance across all forecast horizons. For the most challenging task, the 30-minute ahead forecasts, the model achieved a wind speed Mean Absolute Error (MAE) of 0.78 m/s and a wind direction MAE of 33.06°. Furthermore, the use of Gaussian noise concatenation to both input and label training data yielded the most consistent results. A case study further validated the model’s efficacy, with MAE values below 0.43 m/s for wind speed and between 33.93° and 35.03° for wind direction across different forecast horizons. This approach shows that combining strategically deployed sensor networks with machine learning techniques offers improvements in wind nowcasting for airports in complex environments, possibly enhancing operational efficiency and safety.

## Introduction

Wind speed and direction forecasting is essential for aviation safety and operational efficiency, particularly in short-term scenarios where rapid changes impact flight operations [1, 2], presenting substantial prediction challenges, especially in regions with complex topography [3–5] such as Madeira International Airport (LPMA), which demonstrates how geographical features can complicate wind forecasting. Located in an area with complex air currents [6], LPMA operates under strict wind-related constraints, necessitating enhanced predictive capabilities [7]. Traditional forecasting methods often fall short in such environments [8].

Meteorologists have long used Numerical Weather Prediction (NWP) models for weather forecasting. These systems, however, struggle to capture nuanced, rapidly evolving wind patterns crucial for aviation safety, particularly near airports [3, 4]. NWP models’ computational intensity, due to complex mathematical calculations performed on large, gridded data, also limits their applicability to real-time decision-making in dynamic airport environments [9]. New Machine Learning (ML) techniques offer potential weather prediction improvements, capable of efficiently interpreting complex atmospheric patterns [3, 10]. In short-term forecasting or nowcasting, ML not only enhances traditional methods but also presents as a viable alternative, providing insights within a six-hour window [11, 12].

ML has advanced wind forecasting in sectors such as renewable energy [10, 13], but its application to aviation-specific wind nowcasting remains underdeveloped [1, 4]. Recent studies have shown progress in this area. Lawrence et al. [14] achieved a Mean Absolute Error (MAE) of 0.73 m/s for wind speed and an MAE 35° for wind direction predicting wind at airports, using data with a 1-month time resolution and 318 measurements, which is insufficient for typical nowcasting applications. Another study at Grand Canaria Airport [15] achieved an MAE of 1.23 m/s and 15.80° for wind speed and direction, respectively, using 30-minute resolution runway wind measurements. These works represent a step towards airport wind nowcasting, yet opportunities remain for improving temporal resolution and prediction accuracy.

Recent studies have explored data augmentation techniques to improve time series regression model performance. Systematic sampling and augmentation methods have been shown to enhance model accuracy and generalization in 86.67% of the evaluated scenarios [16]. Traditional interpolation techniques applied to economic time series have demonstrated substantial improvements in predictions when used with autoregressive integrated moving average and neural network models [17]. Advanced augmentation methods using autoencoders, variational autoencoders, and Wasserstein generative adversarial networks have outperformed existing techniques, reducing mean absolute errors by 2.23%, 2.73%, and 2.97%, respectively, in electricity price forecasting [18]. A hybrid approach combining time-warping and jittering has shown promising results for short-term time series prediction, improving performance by 16.32% to 42.14% in non-seasonal time series forecasting [19].

In this context, this research proposes a dedicated wind measurement network and a Deep Learning (DL) model for real-time, high-resolution wind forecasts. The system aims to predict wind speed and direction for LPMA’s runway up to 30 minutes ahead with a 1-minute resolution. Using machine learning techniques combined with data augmentation, this approach addresses the limitations of traditional NWP models, which generally update only once or twice daily, and even more frequent NWP nowcasting systems, such as the Met Office’s nowcasting demonstration project [20], which updates every hour. By achieving runtime predictions in under 1 minute, this ML-based model seeks to provide significantly more frequent updates, offering superior temporal resolution and making it highly suitable for applications requiring near-instantaneous nowcasting updates.

This study is structured into four main sections. The first section outlines the methodology, detailing the deployment of a network of wind stations around the airport and the use of collected data, enhanced through augmentation, to train a deep learning model. The second section presents the results, highlighting the performance of the system. In the final sections, the results are analyzed and discussed, leading to the conclusion that the proposed system can generate standalone wind forecasts for the airport runway with higher temporal resolution than traditional methods.

## Methodology

This study involved several key steps. The first step was the deployment of stations for meteorological data collection. The second step was to collect data and generate a 1-minute step time-synchronized database. The next steps evolved configuring and fine-tuning a DL model for improved performance and applying data augmentation techniques to improve the model’s robustness. The final step comprised training and testing the model using the collected dataset.

### Station network deployment

**Fig 1:**
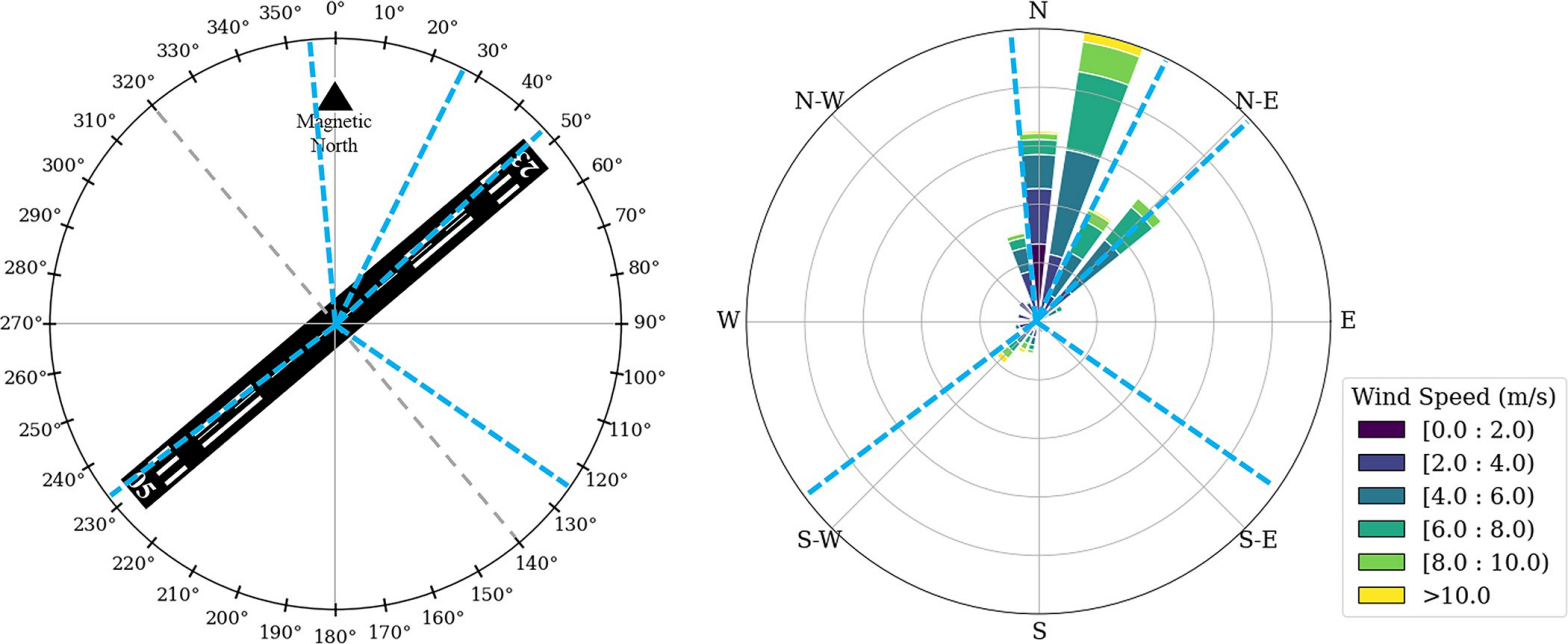
Azimuthal directions of station locations relative to the airport (blue dashed lines) and wind rose for LPMA (2001–2024). Runway 05 and runway 23 are on opposite sides.

**Fig 2:**
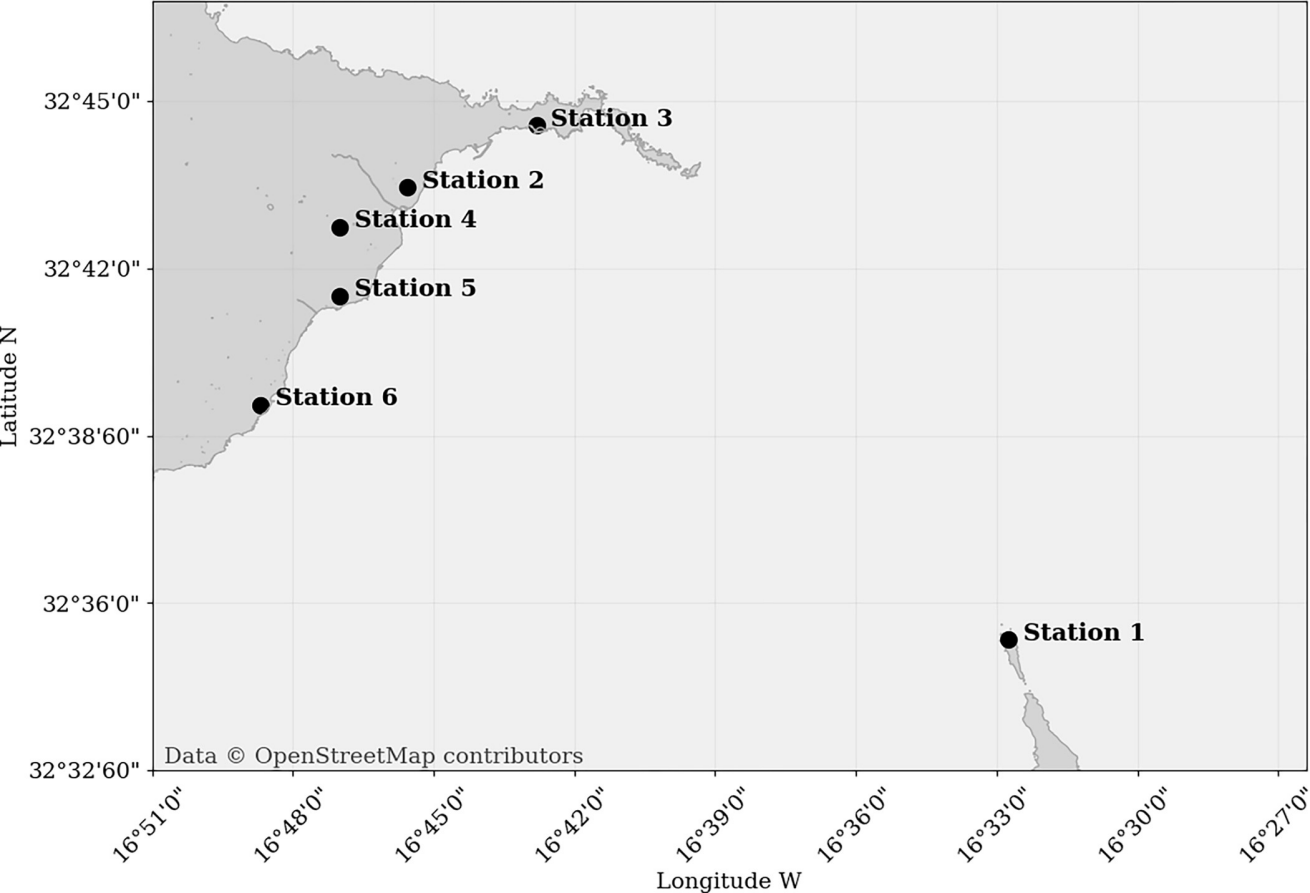
Map showing the location of the station network with five stations in the vicinity to the airport (2,3,4), one at the airport (5) and one and one at the nearby island (1).

An analysis of wind conditions was conducted using data spanning from 2001 to 2024 to determine the optimal location of each station. This analysis utilized Meteorological Aerodrome Report (METAR) data from the LPMA airport, where wind speed and direction were reported as 10-minute averages recorded at 30-minute intervals. Data was sourced from Iowa State University’s Iowa Environmental Mesonet (IEM). The stations were installed according to available terrain locations to cover the most prevalent wind directions. The network was positioned to encircle the airport, enabling comprehensive monitoring of wind conditions in the vicinity. Fig 1 presents a wind rose for LPMA based on 23 years of data, along with the azimuthal directions of station locations relative to the airport. Fig 2 shows the geographic location of the full station network.

Six stations were strategically placed at the following global positioning system coordinates: Station 1 (32°35′20.71″ N, 16°32′44.65″ W) is located southeast of the airport; Station 2 (32°43′27.13″ N, 16°45′33.23″ W) lies to the north-northeast; Station 3 (32°44′33.86″ N, 16°42′48.37″ W) is positioned to the northeast; Station 4 (32°42′44.94″ N, 16°46′59.82″ W) is found to the north-northwest; Station 5 (32°41′30.76″ N, 16°47′0.02″ W) is located at the airport itself; and Station 6 (32°39′32.81″ N, 16°48′41.25″ W) lies to the southwest. From Figs 1 and 2, it is evident that the network successfully covers the predominant winds from the north and northeast, while also capturing wind conditions from the southeast and southwest sectors. Additionally, the network’s positioning ensures coverage of the runway alignment for both runway 05 and runway 23.

### Database

Each station was equipped with a wind speed and direction ultrasonic sensor, model CWT-UWD-SD, manufactured by ComWinTop. All stations were configured to take one measurement every 3-second, synchronized across the entire network to ensure data collection occurred simultaneously. These measurements were transmitted in real-time to a central server using RESTful API and POST methods. Each transmission included a timestamp, a control indicator to monitor the station’s functionality, as well as the wind speed and direction data.

**Fig 3:**
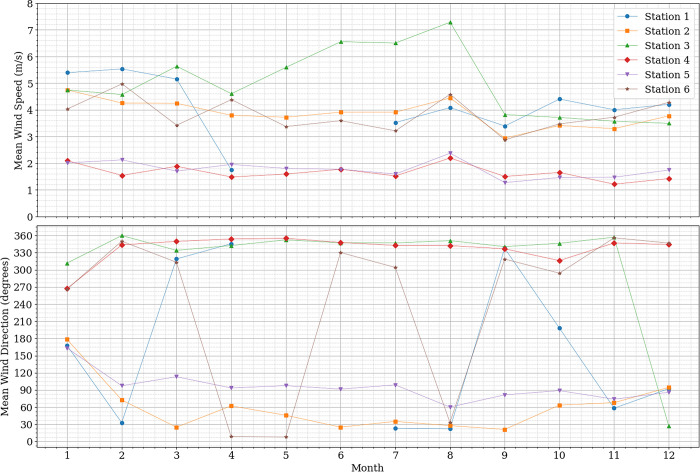
Monthly mean wind direction and speed recorded by each station.

**Fig 4:**
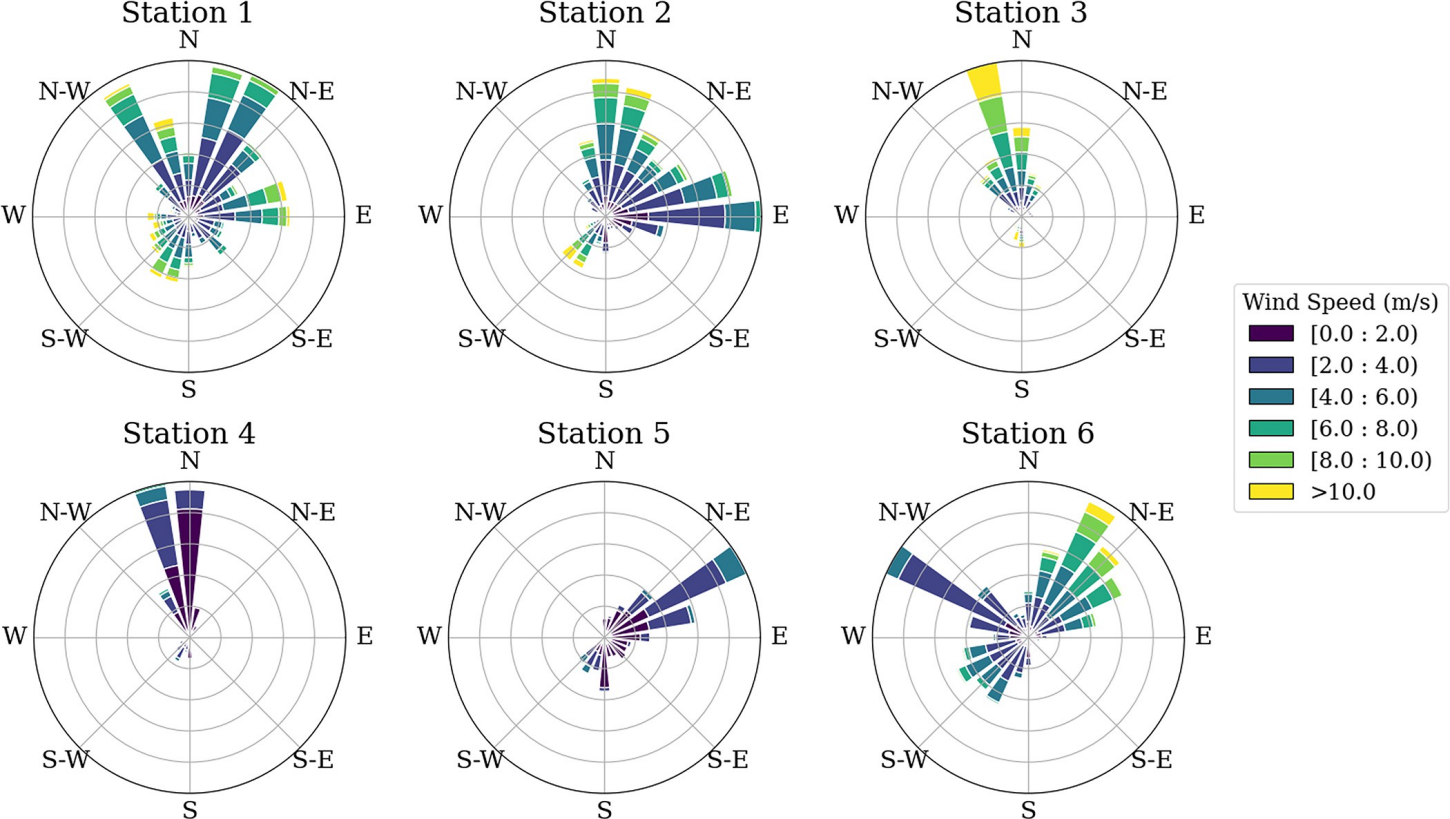
Wind rose diagrams for stations 1 through 6, covering the period from 16 August 2023 to 20 August 2024.

The server, operating on a Linux system, received the real-time data from all stations, processed it, and stored it in a database with the mean value for wind speed and direction at a 1-minute resolution. The final dataset, which was further worked in this research, spans from 16 August 2023 to 20 August 2024. Tables 1 and 2 provide a statistical summary of the database. Fig 3 presents the monthly statistics for each station, while Fig 4 illustrates the wind rose, showing the wind patterns observed across the network. This database has been made available by the authors under the title “Madeira Airport 1-minute Wind Speed and Direction”, in Mendeley Data [21].

**Table 1:** Database general statistics.

Variable	Station 1	Station 2	Station 3	Station 4	Station 5	Station 6
**Mean Wind Speed (m/s)**	4.43	3.87	5.04	1.66	1.78	3.83
**Median Wind Speed (m/s)**	3.89	3.27	4.42	1.35	1.54	3.34
**Max Wind Speed (m/s)**	22.03	23.7	23.89	19.43	14.76	17.39
**Min Wind Speed (m/s)**	0	0	0	0	0	0
**Standard Deviation (Speed)**	2.7	2.62	3.66	1.35	1.35	2.3
**Mean Wind Direction (°)**	26	47	348	341	91	337
**Standard Deviation (Direction)**	1.62	1.31	1.13	1.03	1.2	1.63
**Data Points**	375383	519127	528259	529226	528923	529049

**Table 2:** Database statistics for day (07:00–19:00) and night (19:00–07:00) periods.

Variable	Period	Station 1	Station 2	Station 3	Station 4	Station 5	Station 6
**Mean Wind Speed (m/s)**	**Day**	4.42	3.87	5.02	1.51	2.17	4.02
	**Night**	4.44	3.87	5.06	1.84	1.31	3.6
**Median Wind Speed (m/s)**	**Day**	3.87	3.25	4.5	1.2	1.99	3.58
	**Night**	3.92	3.3	4.33	1.48	0.93	3.15
**Max Wind Speed (m/s)**	**Day**	22.03	23.7	23.89	19.43	14.76	17.39
	**Night**	20.63	23.17	22.19	14.16	11.18	16.96
**Min Wind Speed (m/s)**	**Day**	0	0	0	0	0	0
	**Night**	0	0	0	0	0	0
**Standard Deviation (Speed)**	**Day**	2.65	2.6	3.68	1.33	1.31	2.43
	**Night**	2.76	2.65	3.63	1.35	1.23	2.12
**Mean Wind Direction (°)**	**Day**	29	57	350	337	102	53
	**Night**	24	33	346	345	81	327
**Data Count**	**Day**	205086	288281	288385	288443	288386	288509
	**Night**	170297	230846	239874	240783	240537	240540

### Model architecture and parameters

The model architecture and input time lag were jointly optimized using Bayesian optimization, a probabilistic approach to global optimization of black-box functions that construct a probabilistic model of the objective function [22] and may be used to select the most promising hyperparameters to evaluate in a sequential manner [23]. This method is particularly effective for optimizing computationally expensive functions, such as neural network training. In this study, Bayesian optimization was employed to fine-tune the model’s hyperparameters and to determine the optimal input time lag, searching from a space between -180 and -1, which represents the historical time window of data fed into the model. This optimization approach allowed for efficient exploration of the high-dimensional space of architectural choices and temporal dependencies, aiming to achieve a more robust and accurate predictive model.

The Time-Series Embeddings from Language Models (TELMo) [2] architecture served as a reference and employs a hybrid approach, combining convolutional, recurrent, and dense layers to capture both local and temporal patterns in the input data. The input is first processed through a 1-dimensional convolutional layer with 64 filters and a kernel size of 3, which acts as an embedding layer to extract features. This is followed by two stacked bidirectional LSTM layers, each designed with a hidden state size that is four times larger than the input dimension. This configuration enhances the model’s ability to effectively capture long-term dependencies in the data. By processing sequences in both forward and backward directions simultaneously, the bidirectional nature of these LSTM layers ensures a more comprehensive understanding of contextual relationships within the input. Stacking two such layers further deepen the model’s capacity to learn complex patterns and interdependencies, making it particularly well-suited for tasks requiring nuanced sequence analysis. Projection layers and dropout are applied between LSTM layers to reduce dimensionality and prevent overfitting. The outputs from the embedding layer and both LSTM layers are concatenated and passed through a global max pooling layer to aggregate features across time steps.

**Fig 5:**
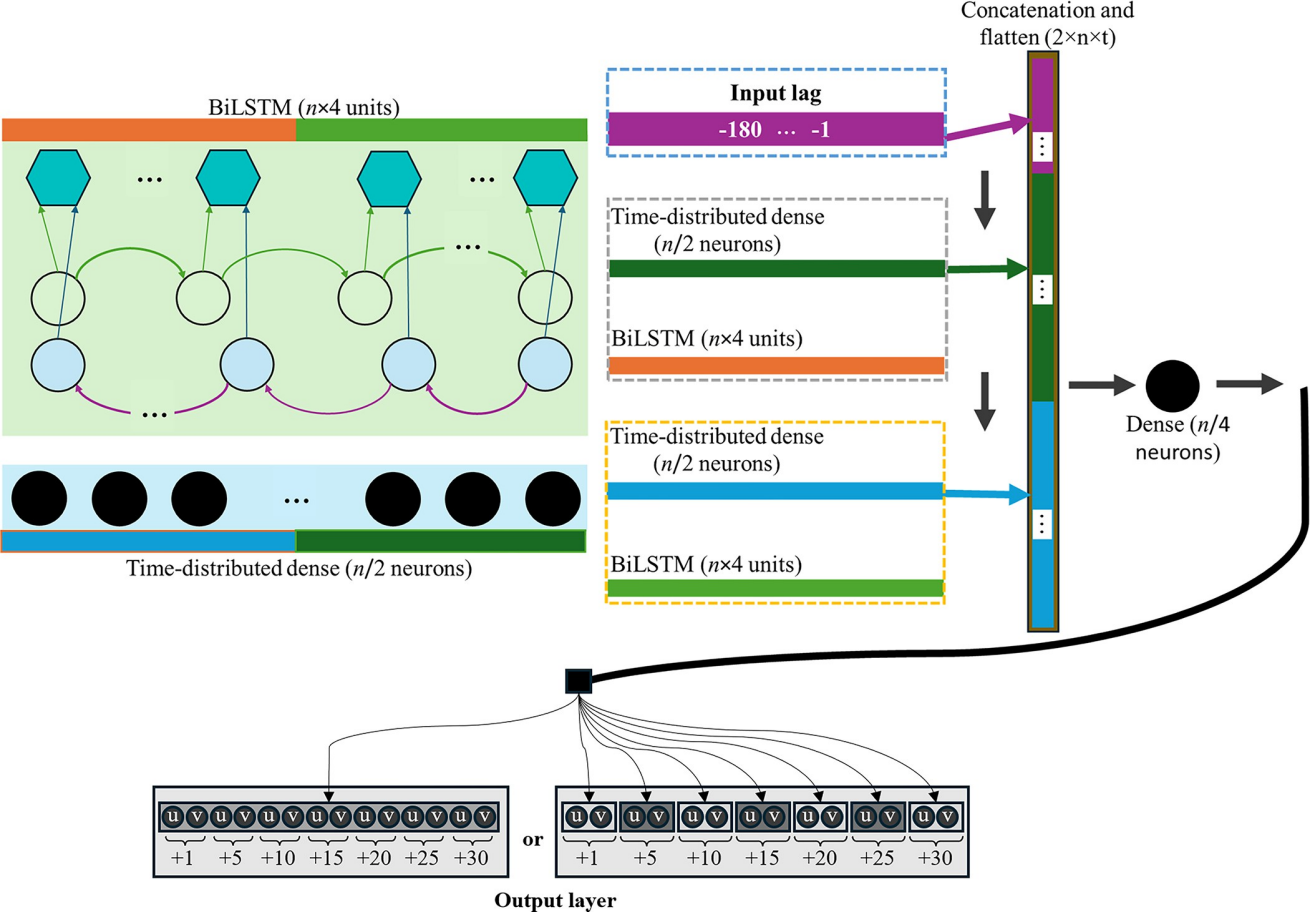
TELMo architecture with output layer and input lag optimization.

For the final architecture optimization, the last layer configuration was evaluated by comparing two distinct setups. Specifically, a single dense layer with one neuron per variable and forecast or an architecture consisting of one dense layer per forecast with one neuron for each variable, running in parallel. These two methods were named respectively single-head and multi-head. This comparison allowed for an in-depth analysis of the influence of different output layer structures on model performance. These dense layers are used to map the pooled features to the desired output dimension, corresponding to the number of future time points and target variables. The output layer architecture optimization and input lag are illustrated in Fig 5, which highlights the output layer architecture included in the optimization algorithm’s search space. Bayesian optimization was performed over 60 iterations for each output architecture, totaling 120 iterations, with the first 40 dedicated to an initial random search, followed by 80 iterations focused on exploring the most promising regions of the search space.

### Data preprocessing

For data preparation, the dataset was first loaded and filtered to exclude missing values, ensuring the use of complete timesteps, defined as intervals where all stations reported valid measurements. The filtered dataset was then split into training, validation, and test sets based on predefined date ranges, specifically, data between 16 August 2023 and 29 February 2024 was allocated for training, data from 01 March 2024 to 01 April 2024 for validation, and data from 10 July 2024 to 20 August 2024 for testing. The dataset was normalized between 0 and 1 for training the model. The target variables of interest were ’sensor5_u’ and ’sensor5_v’, representing, respectably, the u and v wind components at the airport, which were prepared for the model by transforming the data into sequences for predicting up to 30-minute ahead. These steps correspond to time points (minutes ahead) 1, 5, 10, 15, 20, 25, and 30.

### Data augmentation

Augmentation techniques were explored to evaluate their impact on improving the performance and generalization of the DL regression model applied to meteorological time series data. The approach involved evaluating the model’s performance both without augmentation and with the implementation of the following three distinct augmentation strategies individually: Gaussian noise, Magnitude warping, and Fourier transform-based augmentation. These strategies were also tested under several conditions: augmentation applied only to the input data, augmentation applied to both the input data and the target labels, and the use of exclusively augmented data, as well as a combined approach where the augmented data was merged with the original dataset in a concatenation test to evaluate how increased data diversity influences model accuracy and generalization. Augmentation methods were not applied to either the validation or test datasets.

#### Gaussian noise

The Gaussian noise augmentation technique aimed to introduce variability and mimic real-world noise in the input data [24]. This was achieved by adding small random perturbations, drawn from a normal distribution with a standard deviation of 0.01, directly to the time series data. The added noise simulated natural measurement errors or environmental fluctuations that could affect the sensor readings. By training the model on noisy data, the Gaussian augmentation made the model more robust to slight variations or inaccuracies in the input, ensuring that small disturbances in the data did not significantly affect the model’s predictions during inference. Mathematically, for each *x*_*i*_∈*X*

$$x_{i}^{\prime }=x_{i}+N(0,\sigma )$$ (1)

where $x_{i}^{\prime }$ is the augmented value and N(0,σ) represents a normal distribution with mean 0 and standard deviation *σ* [24].

#### Magnitude warping

The magnitude warping augmentation method involved applying a random multiplicative factor to the time series data, effectively distorting the magnitude of the input signals while preserving their overall temporal structure [25]. Specifically, warping factors were drawn from a normal distribution with a mean of 1.0 and a standard deviation of 0.2 and applied independently to each time step across all features. This process introduced non-linear scaling variations along the time axis, simulating scenarios where the intensity or amplitude of the signals could change over time. By introducing these controlled distortions, magnitude warping helped the model become more adaptable to fluctuations in signal strength, improving its generalization to different magnitudes of sensor readings in unseen data. Mathematically, for each *x*_*i*_∈*X*

$$x_{i}^{\prime }=x_{i}\times N(1.0,\sigma )$$ (2)

where $x_{i}^{\prime }$ is the augmented value and N(1.0,σ) represents a normal distribution with mean 0 and standard deviation *σ* [24, 26].

#### Fourier transform

In the Fourier transform-based augmentation method, the input time series data was first transformed into the frequency domain using the Fast Fourier Transform (FFT) [27, 28]. This process converts the temporal data into its constituent frequencies, allowing for manipulation of the signal in the frequency domain. After the transformation, the frequency components were randomly amplified within a range of 0.9 to 1.1, introducing subtle variations in the periodic characteristics of the data while maintaining the overall structure of the original signal. Once the frequency components were modified, an Inverse Fourier Transform (IFFT) was applied to convert the data back into the time domain, resulting in a new time series with altered frequency characteristics. This technique helps simulate natural variations in the underlying periodic patterns, making the model more resilient to variations in the temporal dynamics of the data during the prediction phase. For this augmentation technique, the FFT was applied to obtain frequency domain representation

F=FFT(X) (3)

then the frequency components multiplied by a random factor *r*∈[0.9,1.1]

$$F^{\prime }=F\times r$$ (4)

and the inverse FFT applied to return to the time domain:

$$X^{\prime }=\mathrm{IFFT}(F^{\prime })$$ (5)

where *F* is the Fourier transform of *X*, *r* is a random scaling factor applied to each frequency component and *X*′ is the time series after the modification [27, 28].

### Training and evaluation

The model was compiled with the Adam optimizer and a Mean Squared Error (MSE) loss function. To prevent overfitting, early stopping was applied by monitoring the validation loss with a patience of 10 epochs, while the best model weights were restored once training concluded. The training process was performed for 100 epochs with a batch size of 512, using the training dataset and validated against a separate validation set. To ensure robust results and account for variability, the model was trained 30 times, which is the point at that the central limit theorem begins to apply [29]. This theorem asserts that, regardless of the underlying population distribution, the distribution of sample means will approximate a normal distribution, provided the sample size is sufficiently large [29]. For model evaluation, MAE, MSE and the Root Mean Squared Error (RMSE) were used as performance metrics, calculated using

$$MAE=\left\{ \begin{array}{c} \frac{1}{m}\sum_{i=1}^{m}|Y_{i}-{\hat{Y}}_{i}|,if\;Y_{i},{\hat{Y}}_{i}\;in\;m/s\\ \frac{1}{m}\sum_{i=1}^{m}|\left(Y_{i}-{\hat{Y}}_{i})-360\times \lfloor \frac{Y_{i}-{\hat{Y}}_{i}+180}{360}\rfloor \right|,if\;Y_{i},{\hat{Y}}_{i}\;in\;degrees \end{array}\right.$$ (6)

$$MSE=\left\{ \begin{array}{c} \frac{1}{m}\sum_{i=1}^{m}{\left(Y_{i}-{\hat{Y}}_{i}\right)}^{2},if\;Y_{i},{\hat{Y}}_{i}\;in\;m^{2}/s^{2}\\ \frac{1}{m}\sum_{i=1}^{m}{\left(\left(Y_{i}-{\hat{Y}}_{i})-360\times \lfloor \frac{Y_{i}-{\hat{Y}}_{i}+180}{360}\right\rfloor \right)}^{2},if\;Y_{i},{\hat{Y}}_{i}\;in\;degrees \end{array}\right.$$ (7)

$$RMSE=\left\{ \begin{array}{c} \frac{1}{m}\sum_{i=1}^{m}\sqrt{{\left(Y_{i}-{\hat{Y}}_{i}\right)}^{2}},if\;Y_{i},{\hat{Y}}_{i}\;in\;m/s\\ \frac{1}{m}\sum_{i=1}^{m}\sqrt{{\left(\left(Y_{i}-{\hat{Y}}_{i})-360\times \lfloor \frac{Y_{i}-{\hat{Y}}_{i}+180}{360}\right\rfloor \right)}^{2}},if\;Y_{i},{\hat{Y}}_{i}\;in\;degrees \end{array}\right.$$ (8)

were *Y*_*i*_ represents the actual values, $\hat{Y_{i}}$ represents the predicted values, $\bar{Y}$ is the mean of the actual values, and *m* is the number of observations [2, 30, 31].

The data used for training, validation, and testing was normalized to a range between 0 and 1 using the MinMaxScaler() function. To address the limitation posed by the temporal span of the dataset and enable evaluation of the model under varying seasonal conditions, a parallel training procedure was implemented. In this approach, the data from March 1, 2024, to April 1, 2024, originally part of the validation set, was reassigned as the test set representing a winter season, while the data from July 10, 2024, to August 20, 2024, was used for validation representing the summer season. This adjustment ensured that the model was tested on unseen data corresponding to a distinct seasonal period, thereby facilitating its evaluation under diverse seasonal conditions.

## Results

This section presents and discusses the outcomes of model optimization, data augmentation strategies, and the performance of the final model.

### Optimization

**Fig 6:**
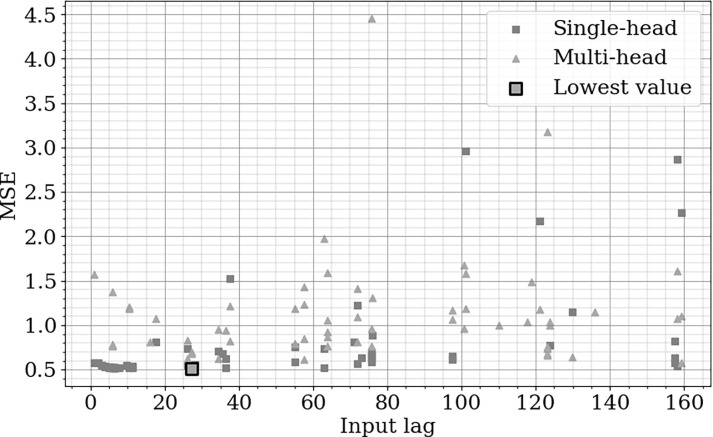
Optimization search space and MSE performance across input lags for single-head and multi-head configurations.

Fig 6 illustrates the optimization space and corresponding results obtained through Bayesian optimization, highlighting the key configurations evaluated throughout the process. The figure provides a visual representation of the performance across varying input lags, allowing for the identification of the most effective model parameters.

In Fig 6, a clear trend emerges where lower MSE values are concentrated at input lags below 40. The single-head output demonstrates more consistent performance in this range, while the multi-head output exhibits greater variability, particularly at higher input lags. The optimal input lag was identified at 27-minute for the single-head output, which achieved a minimum MSE of 0.51.

The final model configuration was selected following these results, using a single dense layer with 14 neurons, and an input lag of 27-minute. This configuration was chosen to maximize model efficiency while minimizing error, as evidenced by the observed MSE results.

### Data augmentation

**Fig 7:**
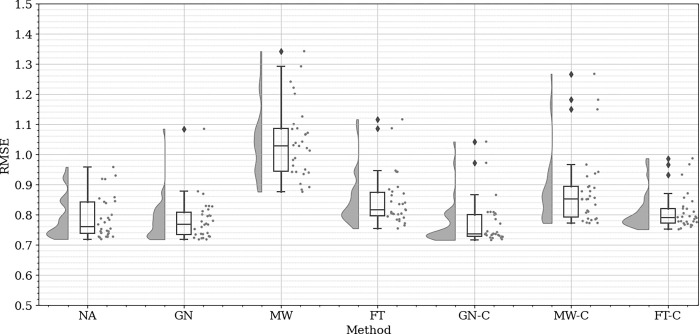
Comparative analysis of data augmentation techniques based on RMSE values.

Fig 7 presents the results obtained from applying the data augmentation techniques. These techniques are No Augmentation (NA), Gaussian Noise (GN), Magnitude Warping (MW), Fourier Transform (FT), Gaussian Noise Concatenated (GN-C), Magnitude Warping Concatenated (MW-C), and Fourier Transform Concatenated (FT-C). The figure provides a comparative analysis of these methods, highlighting their respective impacts on the model’s performance.

**Fig 8:**
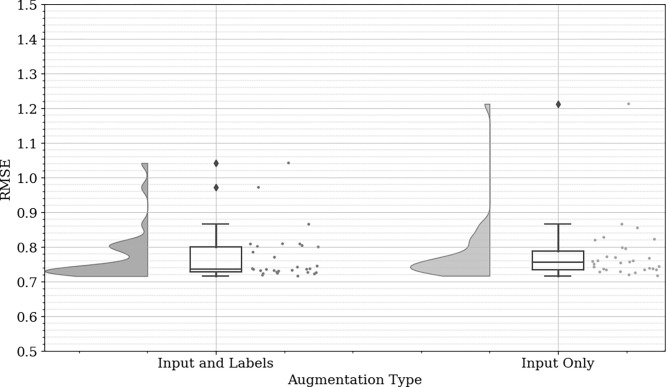
Comparative analysis of GN-C augmentation MSE when applied to input alone versus both input and labels.

A noticeable trend is the superior performance of the concatenated techniques (GN-C, MW-C, and FT-C) when compared to their non-concatenated counterparts. Among these, GN-C achieves the best results, displaying the lowest median RMSE and the narrowest interquartile range. This indicates that GN-C not only improves predictive accuracy but also reduces variability, likely due to the more effective introduction of noise combined with concatenation, which enhances data diversity without compromising its underlying structure. In Fig 8, the results of the GN-C with augmented input and labels versus augmented input only are depicted. The augmentation of both input and labels yields a more consistent RMSE distribution, characterized by a reduced presence of outliers and a narrower interquartile range when compared to the input only augmentation method. This observation suggests that augmenting both input and labels concurrently enhances the model’s stability, likely contributing to improved generalization across diverse data variations. Consequently, the final model was trained using augmented input and labels in the training dataset, employing the GN-C technique.

### Final model performance

For the final model evaluation, the test dataset, from 10 July 2024 to 20 August 2024, was used as it provides a realistic scenario with unseen data, ensuring that no information from the test set was introduced during any phase of model development. This dataset, reserved exclusively for testing purposes, allows for a true assessment of the model’s ability to generalize to real-world conditions. It was not involved in training, fine-tuning, or validation, making it an effective benchmark for evaluating the final performance of the deep learning model. This separation reinforces the integrity of the results, ensuring that the model’s predictive power is based on its capacity to handle novel data without overfitting to the patterns present in the training or validation phases.

**Fig 9:**
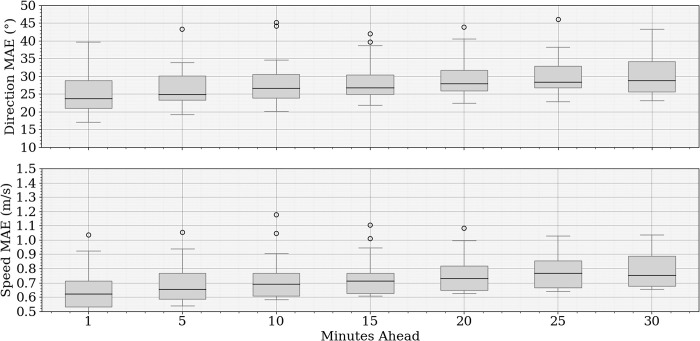
MAE progression for wind direction and speed forecasts at different time horizons.

Fig 9 presents the MAE over the forecast time, showing a gradual increase in errors for both wind direction and speed as the prediction horizon extends. Both the median and variability of the errors increase, indicating an increasing challenge for the model as the forecast lengthens.

Table 3 provides a comprehensive summary of the forecast error metrics for all prediction horizons and variables, including the u and v wind components, wind speed, and direction. These metrics are presented for each prediction interval from 1 to 30-minutes ahead, including their standard deviations.

**Table 3:** Forecast error metrics (mean ± standard deviation) for all timesteps ahead and variables for the test dataset.

minutes ahead	u			v			Speed			Direction		
	MSE	MAE	RMSE	MSE	MAE	RMSE	MSE	MAE	RMSE	MSE	MAE	RMSE
**1**	0.45±0.21	0.52±0.13	0.66±0.13	0.56±0.25	0.57±0.14	0.73±0.15	0.74±0.30	0.65±0.15	0.85±0.16	3764.94±1986.58	29.96±8.02	59.43±15.28
**5**	0.51±0.22	0.55±0.12	0.70±0.13	0.63±0.19	0.60±0.11	0.78±0.12	0.84±0.29	0.70±0.13	0.90±0.15	3687.02±1610.34	30.55±7.03	59.33±12.94
**10**	0.56±0.22	0.57±0.12	0.74±0.12	0.70±0.29	0.64±0.14	0.83±0.14	0.90±0.32	0.72±0.14	0.93±0.15	3745.96±2181.92	31.76±9.12	59.27±15.26
**15**	0.60±0.21	0.59±0.12	0.76±0.12	0.72±0.19	0.65±0.10	0.84±0.10	0.93±0.28	0.74±0.13	0.95±0.14	3494.24±1394.98	31.29±6.64	58.01±11.39
**20**	0.64±0.22	0.61±0.12	0.79±0.12	0.78±0.18	0.67±0.09	0.88±0.10	0.98±0.28	0.76±0.12	0.98±0.14	3577.49±1355.38	32.20±6.47	58.80±10.96
**25**	0.68±0.24	0.63±0.12	0.82±0.13	0.82±0.17	0.69±0.08	0.90±0.09	1.02±0.27	0.77±0.11	1.00±0.13	3603.62±1355.97	32.87±6.50	59.01±11.04
**30**	0.69±0.21	0.63±0.11	0.82±0.11	0.87±0.20	0.71±0.10	0.93±0.10	1.05±0.27	0.78±0.11	1.01±0.13	3676.92±1521.36	33.06±6.95	59.43±12.07

**Fig 10:**
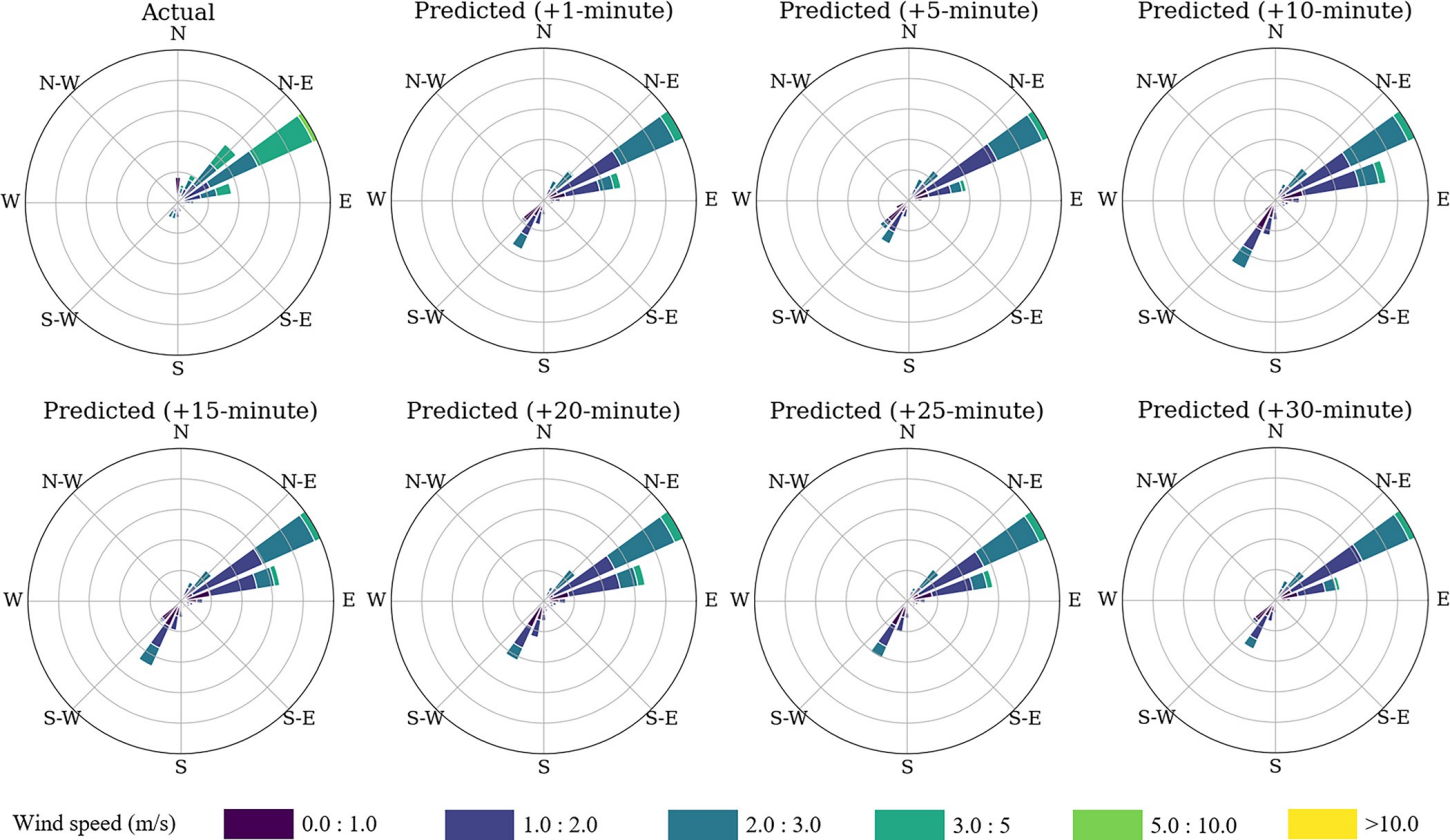
Wind roses for all timesteps ahead for the test dataset.

The best results were observed at the 1-minute prediction horizon, where both wind speed and direction errors were minimized. The model achieved a speed MAE of 0.65 m/s and a direction MAE of 29.96°, indicating relatively high accuracy at short-term forecasts. Although the prediction error increases as the forecast horizon extends, these results demonstrate the model’s strong capability for immediate short-term wind nowcasting. The forecasts depicted as wind roses for all timesteps ahead are shown in Fig 10, showing that the model can follow the wind patterns at all prediction intervals.

**Fig 11:**
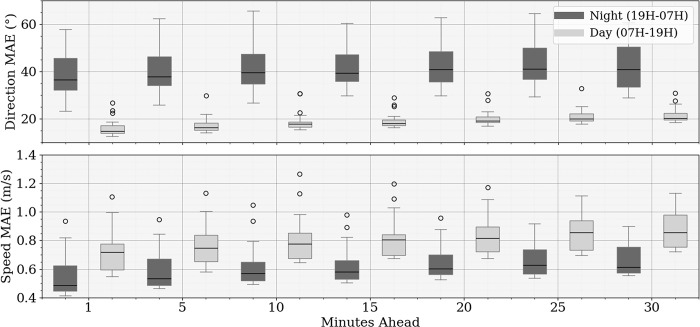
Progression of MAE for wind direction and speed forecasts during nighttime and daytime hours.

In Fig 11, the model’s performance is further analyzed by comparing the MAE for both wind direction and speed during night (19H-07H) and day (07H-19H) across all prediction horizons. The results clearly indicate a trend where nighttime predictions exhibit higher errors in wind direction, with MAE exceeding 40° for all horizons, compared to significantly lower errors during the day In contrast, wind speed predictions perform better at night, showing consistently lower MAE values compared to daytime predictions. This distinction between night and day accuracy highlights the model’s sensitivity to diurnal variations, likely influenced by changes in atmospheric stability and turbulence patterns.

This distinction may arise from more challenging atmospheric conditions during the nighttime, such as increased turbulence or variability, which have a greater impact on wind direction, making it more difficult for the model to provide accurate predictions.

The overall performance of the model across different seasons, including both winter and summer periods, is summarized in Table 4.

**Table 4:** Summary of overall model performance (mean ± standard deviation) across different seasons.

Season	MSE	MAE	RMSE	R2
**Summer**	0.66±0.18	0.62±0.09	0.80±0.10	0.64±0.09
**Winter**	0.62±0.09	0.58±0.06	0.78±0.05	0.71±0.04

The seasonal analysis included a validation strategy where the test and validation datasets were interchanged, aiming to assess the model’s performance across different temporal conditions. The model demonstrated robust performance across different seasonal periods, as evidenced in Table 4. The results suggest that the model maintained consistent predictive capabilities regardless of the seasonal variations in the input parameters, confirming its reliability for year-round applications.

#### Case studies

**Fig 12:**
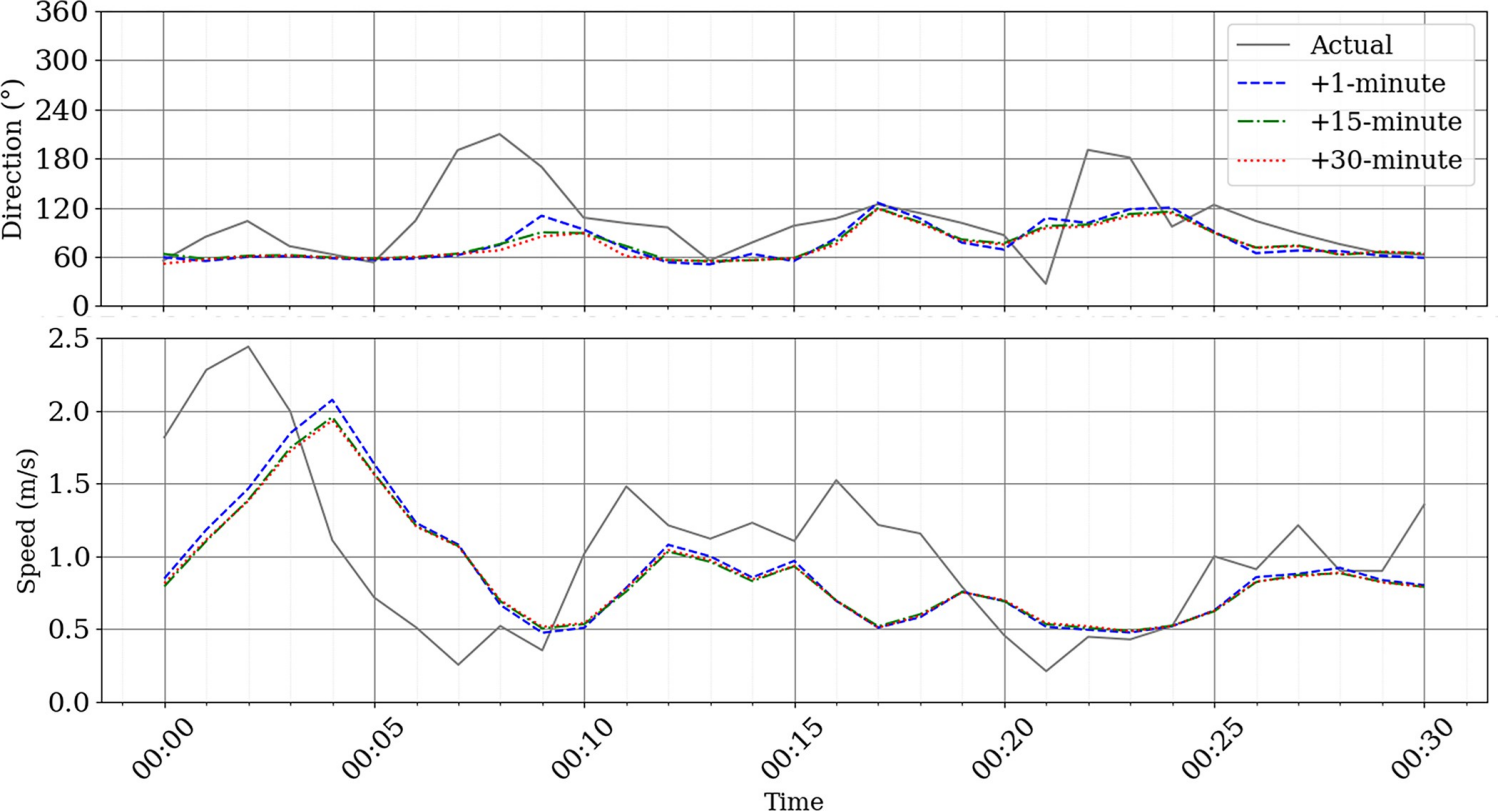
High resolution wind speed and direction nowcast on 13 July 2024 between 00:00 UTC and 00:30 UTC.

To thoroughly evaluate the model’s performance at a high resolution, two case studies were conducted. The first covered the time interval between 00:00 and 00:30 on July 13, 2024, and the second spanned from 21:30 to 22:00 on August 10, 2024. Each interval includes 30 data points, sampled at a 1-minute resolution. Fig 12 illustrates the wind speed and direction forecasts for lead times of 1 minute, 15 minutes, and 30 minutes during the first case study.

In the period of analysis for this case study, the model’s performance was consistent with the results obtained from the full test dataset, particularly demonstrating robust performance in predicting wind speed. The error metrics for wind speed prediction over different forecast horizons highlight the model’s accuracy. At the 1-minute forecast horizon, the MAE was 0.42, the MSE was 0.30, and the RMSE was 0.55. For the 15-minute and 30-minute forecast horizons, the performance remained stable with an MAE of 0.43, MSE of 0.31, and RMSE of 0.55 at 15 minutes, and an MAE of 0.43, MSE of 0.30, and RMSE of 0.55 at 30 minutes.

Regarding wind direction, while the model showed good predictive capacity, the inherent variability in wind direction data resulted in slightly higher error values. For the 1-minute forecast horizon, the model achieved an MAE of 34.02, an MSE of 2322.53, and an RMSE of 48.19. Similar values were observed for the 15-minute and 30-minute horizons, with MAE values of 33.93 and 35.03, MSE of 232.78 and 2486.22, and RMSE values of 48.30 and 49.86, respectively. Despite the increased complexity of predicting wind direction, these results highlight the model’s overall reliability across both wind speed and direction forecasting tasks, even at such high temporal resolution.

**Fig 13:**
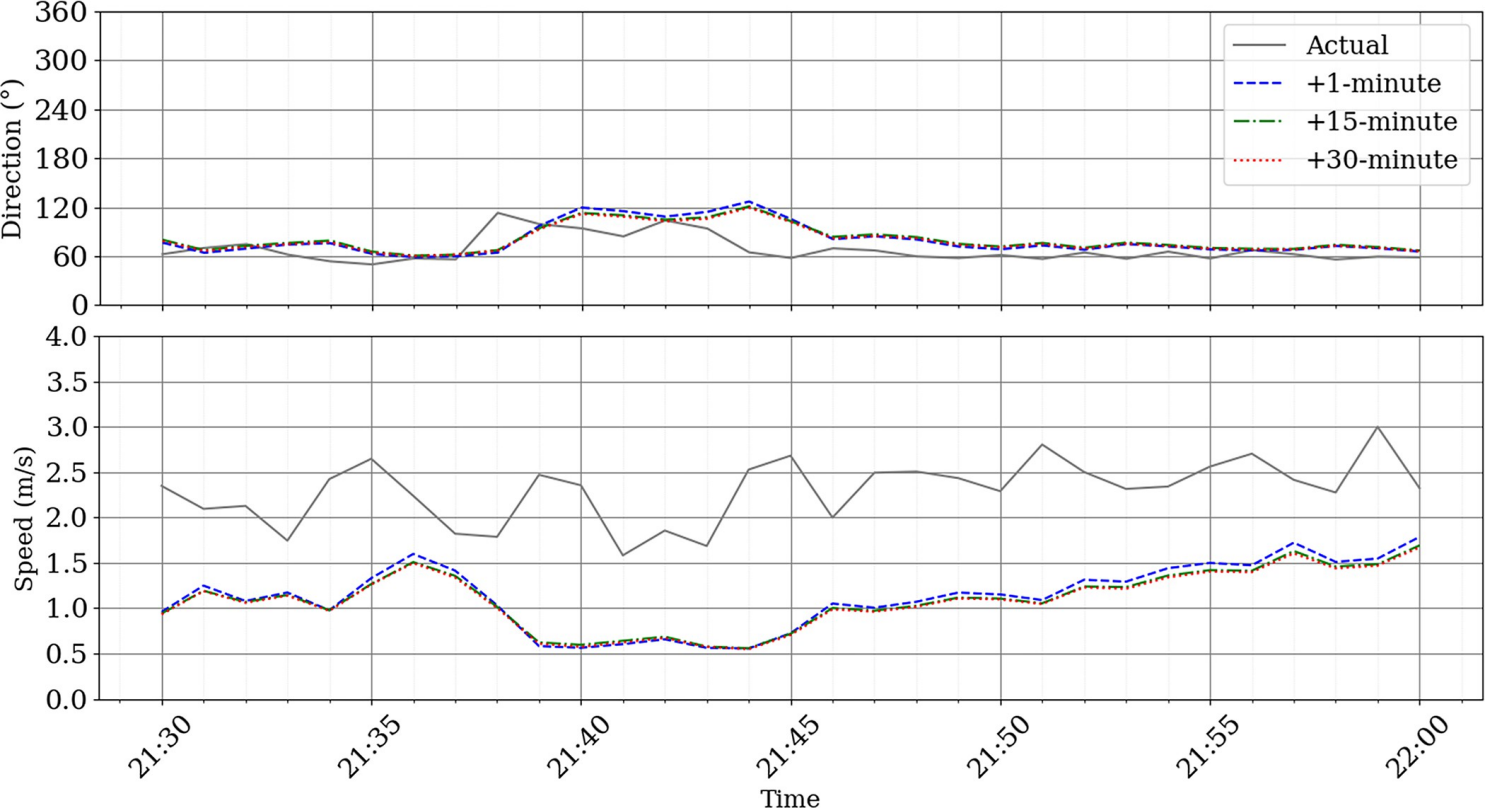
High resolution wind speed and direction nowcast on 10 August 2024 between 21:30 UTC and 22:00 UTC.

Fig 13 provides a detailed visualization of the wind speed and direction forecasts for the second case study, covering the period from 21:30 to 22:00 on August 10, 2024. Similar to the first case study, this interval consists of 30 data points at a 1-minute resolution. The figure compares forecasts at lead times of 1 minute, 15 minutes, and 30 minutes, offering insights into the model’s performance in capturing wind dynamics during this specific time frame.

For wind speed in the second case study, the model showed slightly higher errors compared to the first case study. At a 1-minute lead time, the MAE was 1.17, the MSE was 1.53, and the RMSE was 1.24. For the 15-minute lead time, the MAE was 1.20, the MSE was 1.60, and the RMSE was 1.27. At the 30-minute lead time, the MAE was 1.22, the MSE was 1.63, and the RMSE was 1.28.

For wind direction, the errors in the second case study were smaller than in the first. At a 1-minute lead time, the MAE was 15.69, the MSE was 451.92, and the RMSE was 21.26. For the 15-minute lead time, the MAE was 15.98, the MSE was 424.17, and the RMSE was 20.60. At the 30-minute lead time, the MAE was 15.16, the MSE was 391.33, and the RMSE was 19.78.

## Conclusion

This study presents a novel approach to wind nowcasting for aviation applications, using LPMA as a case study, an environment characterized by complex wind patterns. By leveraging a strategically deployed network of six meteorological stations and employing advanced deep learning techniques, a high-resolution wind forecasting model was developed, capable of predicting wind speed and direction up to 30-minute ahead with 1-minute temporal resolution.

The optimized model architecture, featuring a single dense layer with 14 neurons and an input lag of 27 minutes, demonstrated robust performance across various prediction horizons. For immediate short-term forecasts (1-minute ahead), the model achieved a wind speed MAE of 0.65 m/s and a wind direction MAE of 29.96°. Although prediction errors increased with longer forecast horizons, the model maintained commendable accuracy even at the 30-minute mark, with a speed MAE of 0.78 m/s and a direction MAE of 33.06°.

In comparison to previous studies, there is a significant improvement in accuracy. For instance, the wind speed MAE shows an approximately 11% reduction compared to the 0.73 m/s MAE reported by Lawrence et al. [14], while utilizing data with a much higher temporal resolution (1-minute versus 1-month). Additionally, the wind direction MAE is reduced by about 14% compared to their 35°. Furthermore, relative to the study conducted at Grand Canaria Airport [15], the wind speed MAE demonstrates a 37% reduction, highlighting the enhanced precision achieved with this optimized model

Exploring diverse data augmentation techniques revealed that Gaussian Noise Concatenation technique applied to both input and labels yielded the most consistent and accurate results. This finding highlights the importance of data diversity and noise simulation in enhancing model robustness and generalization capabilities.

A case study conducted for a 30-minute period on 13 July 2024 further validated the model’s efficacy, demonstrating stable performance across different forecast horizons. Consistent accuracy was observed in wind speed predictions, with MAE values below 0.43 m/s across all tested forecast steps. While wind direction predictions showed slightly higher variability, the model achieved respectable MAE values ranging from 33.93° to 35.03° across the forecast horizons. A second case study on 10 August 2024 revealed slightly higher wind speed errors, with MAE values between 1.17 m/s and 1.22 m/s, but improved wind direction accuracy, with MAE values ranging from 15.16° to 15.98° across forecast horizons.

These results represent a significant advancement in short-term wind forecasting for aviation applications, particularly in regions with complex topography. According to the International Civil Aviation Organization (ICAO), the recommended accuracy for wind forecasts in aeronautics specifies a wind speed error of ±2.5 m/s (5 kt) and a directional error of 20° in at least 80% of cases [32]. The proposed system achieved a directional error of 20.60° and a wind speed error of 0.53 m/s in 80% of 1-minute resolution forecasts, aligning with the ICAO recommendations for direction and significantly surpassing the standards for wind speed. These advancements offer substantial potential for improving operational efficiency and safety at LPMA and other airports with similarly challenging environments.

Nevertheless, certain limitations of the study must be acknowledged. The model’s performance was evaluated over a specific period, and longer-term studies may be necessary to assess its robustness across different weather patterns. Moreover, while the model shows promise, its integration into existing aviation weather systems and decision-making processes warrants further investigation.

Future research directions could include expanding the station network to capture more nuanced wind patterns and exploring the model’s applicability to other airports with complex wind conditions. Additionally, investigating the potential of transfer learning techniques could facilitate the adaptation of this model to different geographical locations with minimal retraining.
